# Aquaporin-4 Antibodies Are Not Related to HTLV-1 Associated Myelopathy

**DOI:** 10.1371/journal.pone.0039372

**Published:** 2012-07-10

**Authors:** Felipe von Glehn, Sven Jarius, Augusto C. Penalva de Oliveira, Carlos Otávio Brandão, Alessandro S. Farias, Alfredo Damasceno, Jorge Casseb, Adriel S. Moraes, Ana Leda F. Longhini, Klaus-Peter Wandinger, Benito P. Damasceno, Brigitte Wildemann, Leonilda M. B. Santos

**Affiliations:** 1 Neuroimmunology Unit, Department of Genetics, Evolution and Bioagents, University of Campinas, Campinas, Brazil; 2 Department of Neurology, University of Campinas, Campinas, Brazil; 3 Division of Molecular Neuroimmunology, Department of Neurology, University of Heidelberg, Heidelberg, Germany; 4 Neuroinfectious Disease Unit, Department of Internal Medicine, University of Campinas, Campinas, Brazil; 5 Department of Neurology, Emilio Ribas Institute of Infectious Diseases, Sao Paulo, Brazil; 6 Institute for Experimental Immunology, affiliated to Euroimmun, Luebeck, Germany; Julius-Maximilians-Universität Würzburg, Germany

## Abstract

**Introduction:**

The seroprevalence of human T-cell leukemia virus type 1 (HTLV-1) is very high among Brazilians (∼1∶200). HTLV-1 associated myelopathy or tropical spastic paraparesis (HAM/TSP) is the most common neurological complication of HTLV-1 infection. HAM/TSP can present with an acute/subacute form of longitudinally extensive myelitis, which can be confused with lesions seen in aquaporin-4 antibody (AQP4-Ab) positive neuromyelitis optica spectrum disorders (NMOSD) on MRI. Moreover, clinical attacks in patients with NMOSD have been shown to be preceded by viral infections in around 30% of cases.

**Objective:**

To evaluate the frequency of AQP4-Ab in patients with HAM/TSP. To evaluate the frequency of HTLV-1 infection in patients with NMOSD.

**Patients and Methods:**

23 Brazilian patients with HAM/TSP, 20 asymptomatic HTLV-1+ serostatus patients, and 34 with NMOSD were tested for AQP4-Ab using a standardized recombinant cell based assay. In addition, all patients were tested for HTLV-1 by ELISA and Western blotting.

**Results:**

20/34 NMOSD patients were positive for AQP4-Ab but none of the HAM/TSP patients and none of the asymptomatic HTLV-1 infected individuals. Conversely, all AQP4-Ab-positive NMOSD patients were negative for HTLV-1 antibodies. One patient with HAM/TSP developed optic neuritis in addition to subacute LETM; this patient was AQP4-Ab negative as well. Patients were found to be predominantly female and of African descent both in the NMOSD and in the HAM/TSP group; Osame scale and expanded disability status scale scores did not differ significantly between the two groups.

**Conclusions:**

Our results argue both against a role of antibodies to AQP4 in the pathogenesis of HAM/TSP and against an association between HTLV-1 infection and the development of AQP4-Ab. Moreover, the absence of HTLV-1 in all patients with NMOSD suggests that HTLV-1 is not a common trigger of acute attacks in patients with AQP4-Ab positive NMOSD in populations with high HTLV-1 seroprevalence.

## Introduction

Neuromyelitis optica (NMO) is an inflammatory disease of the central nervous system (CNS) of putative autoimmune aetiology, which is characterized by severe attacks of myelitis and optic neuritis (ON) [1], [2]. In 60–80% of cases, NMO is associated with antibodies to aquaporin-4 (AQP4-Ab), the most abundant water channel in the CNS [3]–[4]. AQP4-Ab are also detectable in around 60% of patients with isolated longitudinally extensive transverse myelitis (LETM) [5] and in 5–25% of patients with recurrent, isolated ON [6]–[8], which are therefore considered *formes frustes* of NMO. NMO, LETM, and ON are often referred to as ‘NMO spectrum disorders’ (NMOSD) [9].

It is estimated that 15 to 20 million individuals are infected with the human T-cell leukemia virus type 1 (HTLV-1) worldwide [10]. HTLV-1 infection remains asymptomatic in the vast majority of cases, yet less than 5% of affected individuals will develop two major diseases: adult T-cell leukaemia/lymphoma (ATL) and HTLV-1 associated myelopathy or tropical spastic paraparesis (HAM/TSP) [11]. While HAM/TSP’s pathogenesis is not fully understood, it is thought to be related to a high HTLV-1 provirus burden and an exaggerated proinflammatory cellular immune response, leading to a chronic extensive myelitis [12]. Some case reports have described an acute variant of HAM/TSP, characterized by longitudinally extensive transverse myelitis (LETM) on magnetic resonance imaging (MRI), a key feature of neuromyelitis optica (NMO), which may or may not be associated with ON [13]–[16].

There are few population-based epidemiological studies of NMOSD, but it seems that the disease is more prevalent in peoples of Asian, African-American or Hispanic background when compared with those of Northern European descent [17]–[19]. Accordingly, the proportion of NMOSD patients among all patients with CNS demyelinating disorders is high in Brazil [20]. At the same time, Brazil is among the countries with the highest prevalence of HTLV-1 infected individuals [12], [21]. Moreover, both among patients with AQP4-Ab positive NMOSD and among patients with HAM/TSP an afrodescendant predilection was reported [12], [22]–[24].

As testing for aquaporin-4 antibodies (AQP4-Ab) became available only few years ago, cases of AQP4-Ab positive LETM occurring in the context of HTLV-1 seropositivity might thus have been misdiagnosed as acute HAM/TSP in a subset of patients in the past. Furthermore, AQP4-Ab positive NMO was shown to be frequently preceded by viral or bacterial infections and HTLV-1 infection may act as a trigger of NMO in some cases [1]–[2].

This study aimed to determine the seroprevalence of antibodies to AQP4 in patients with HTLV-1 associated myelopathy (HAM/TSP) and that of HTLV-1 antibodies in patients with neuromyelitis optica spectrum disorders (NMOSD) and to compare the clinical characteristics of a HAM/TSP and NMOSD in Brazilian patients.

## Patients and Methods

### Patients

This is a cross-sectional study that included along with regular visits NMOSD patients who were followed-up at the neurological outpatient unit of the University of Campinas (UNICAMP) Hospital as well as HTLV-1 seropositive asymptomatic and HAM/TSP patients attending the outpatient clinic at the Emilio Ribas State Reference Institute of Infectious Diseases and at the UNICAMP Hospital, São Paulo, Brazil, during the period of January 2011 to January 2012.

At each appointment, demographic and clinical data were collected and the neurological statuses were evaluated by different scales, including the EDSS [25] and Osame scales [26]. We excluded from the study other causes of transverse myelitis, clinically and radiologically, such as spinal cord compression, infectious myelopathy, including parasitic etiology (e.g. *Schistosoma mansoni*, which is endemic in northeast Brazil) [27], spinal cord ischemia or bleeding, vitamin B12 and folate deficiency, among others. We also excluded patients co-infected with hepatitis C virus (HCV), hepatitis B virus (HBV), human immunodeficiency virus types 1 and 2 (HIV-1/2) and HTLV type 2 in the HTLV-1 seropositive group. We decided to exclude these potentially confounding factors, because these viruses co-infection could interact and transactivate each other, thus altering the clinical presentation of the myelopathy. Moreover, HIV itself may cause vacuolar myelopathy, which shares some neurological traits with HAM/TSP and could turn it difficult to determine which virus was causing the clinical myelopathy [28].

Peripheral blood samples were collected from patients diagnosed with HAM/TSP as defined by World Health Organization (WHO) criteria [29], from asymptomatic individuals with positive HTLV-1 serostatus, from patients diagnosed with NMO spectrum disorders (NMOSD) [30], and from healthy controls (HC) (Table 1). NMO was diagnosed according to Wingerchuk’s revised 2006 criteria without the need for positive AQP4-Ab testing. LETM was defined as acute myelitis with spinal cord lesions extending over three or more vertebral segments on magnetic resonance imaging; the median time between onset of transverse myelitis symptoms and spinal MRI in the LETM group was 7 days (range, 5–25 days). ON was defined as the occurrence of at least two episodes of clinical optic neuritis, with an interval of more than 30 days between them, and of no brain lesions outside the optic nerves.

**Table 1:** Demographic and baseline clinical characteristics of patients and controls.

	Patients #	Age (years)*	Gender F/M	Time from first symptoms (years)*	EDSS**	Osame scale	Aqua4-Ab (%)	CSF Oligoclonal bands (%)***
NMOSD	34	38 (14–76)	26/8	4 (1–17)	4.5 (1–8.5)	3.5 (1–10)	20/34 (59%)	10/34 (29%)
NMO	17	38 (17–63)	15/2	5 (2–17)	5.5 (2.0–8.5)	3 (1–10)	14/17 (82%)	7/17 (41%)
HRS	17	38 (14–76)	11/6	1 (1–9)	3.0 (1.0–8.0)	1 (0–10)	6/17 (35%)	3/17 (18%)
LETM	11	43 (14–76)	8/3	1 (1–3)	5.5 (1.0–8.0)	4 (1–10)	4/11 (36%)	3/11 (27%)
ON	6	32 (16–49)	3/3	5 (1–9)	2.5 (1.0–3.0)	0 (0–0)	2/6 (33%)	0/6 (0%)
HAM/TSP	23	49 (22–83)	16/7	8 (4–21)	6.5 (2.0–7.5)	5 (1–9)	0/23 (0%)	N.d.
HTLV-1+ asymptomatic	20	51.5 (34–72)	17/3	N.a.	N.a.	N.a.	0/20 (0%)	N.a.
Normal controls	23	30 (21–61)	16/7	N.a.	N.a.	N.a.	0/23(0%)	N.a.

* Median (range); **EDSS  =  Expanded disability status scale score;

*** CSF Oligoclonal bands =  Two or more cerebrospinal fluid restricted IgG oligoclonal bands.

HRS  =  high risk syndromes (patients at high risk for conversion into NMO); N.a. =  Not applicable; N.d. =  Not done.

### Ethics Statement

UNICAMP and Emilio Ribas Institute of Infectious Diseases Ethics Committees for Research approved the study and all patients provided informed written consent. On the behalf of the minors/children participants involved in our study, we obtained informed written consent from one of their parents.

### Methods

**Figure 1:**
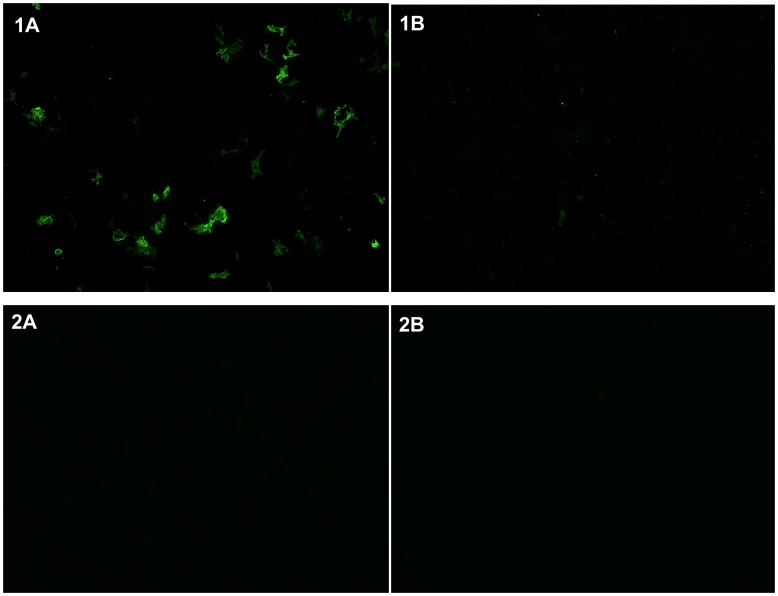
Cell based assay. Antibodies to aquaporin-4 (AQP4) as detected by binding of patient IgG to HEK293 cells transfected with human full length AQP4 (left column) but not to non-transfected control HEK293 cells (right column). **1A and B:** Positive AQP4-Ab test in a patient with NMO according to Wingerchuk’s 2006 criteria [29]. **2A and B:** Negative AQP4-Ab test in a patient with HAM/TSP as defined by World Health Organization criteria [28].

We tested all peripheral blood samples for AQP4-Ab in a standardized cell based immunofluorescence assay (Figure 1) employing recombinant human AQP4 (Euroimmun AG, Germany) [31] at the Neuroimmunology Laboratory of the University of Campinas and for HTLV-1 in a commercial enzyme immunosorbent assay (ELISA) kit (HTLV-I/II ELISA 4.0, MP Diagnostics, Germany). When a serum sample tested positive, it was confirmed by a Western blot (WB) assay (HTLVblot 2.4, MP Diagnostics, Germany) at the Laboratory of Retrovirology of Emilio Ribas Institute of Infectious Diseases. Data were analyzed using GraphPad Prism 5. Statistical significance of differences was determined by Chi-square or Fisher’s exact test for binominal outcomes and by ANOVAs without assuming Gaussian distribution (Kruskal-Wallis test) and subsequent Dunn’s multiple comparison tests. Differences were considered statically significant with *p* values <0.05.

## Results

### Detection of AQP4-Ab in Serum of Patients with NMOSD, HAM/TSP and Healthy Control

Serum AQP4-Ab were detected in 20 out of 34 (59%) patients in the NMOSD group, which included patients with NMO (positive in 14/17 cases) and patients with syndromes considered to confer a high risk for conversion to NMO (recurrent ON or LETM; positive in 6/17 cases) (see Table 1 for details), but in none of the patients previously diagnosed with HAM/TSP (n = 23), in none of the patients with positive HTLV-1 serostatus but no neurological symptoms at the time of presentation (n = 20), and in none of the HCs (n = 23) (p<0.0001; Chi-square test). Moreover, while all patients previously diagnosed with HAM/TSP were positive for HTLV-1 antibodies, as detected by ELISA and confirmed by WB assay, all patients with NMOSD were negative.

During the study, we excluded two asymptomatic patients that turned out to be infected with HTLV type 2 after WB test and three HAM/TSP patients that were co-infected with HCV and/or HIV1/2. All of them tested negative for serum AQP4-Ab.

### Clinical Characteristics and Comparison of the Study Population

The demographic and baseline clinical characteristics of the study population are shown in Table 1. Most patients of the HAM/TSP group had progressive lower limb weakness and muscle spasticity, sensory disturbances, dorsal pain, neurogenic bladder, and bowel and sexual dysfunction. The patients with NMOSD usually presented severe attacks of ON, with poor recovery leading to low vision, with no or only light perception in one or both eyes, and/or myelitis with severe motor disability and deep sense and sphincters disturbance. Pain was also frequent, occurring in 8 out of 11 (73%) in the LETM group and in 12 out of 17 (71%) in the NMO group, and usually affected one or more areas of the chest, waist, legs, and back.

One patient developed an atypical form of HAM/TSP with relapsing subacute LETM and ON. HTLV-1 antibodies were detectable in serum and in the cerebrospinal fluid (CSF) of this patient by western blot analysis; by contrast, serum AQP4-Ab were negative. Moreover, *in vitro* culture of T-cells from this patient’s peripheral blood mononuclear cells (PBMC) revealed an elevated spontaneous proliferative response when compared with a healthy control. After cells were cultured for 48h and pulsed with thymidine during 18h, the proliferative response were determinated by the mean incorporation of thymidine in DNA, and indicated as counts per minute (cpm). This spontaneous proliferation of PBMC *in vitro* is an immunopathologic characteristic of HTLV-1–infected individuals, driven by the HTLV-1-encoded TAX protein and indicate the exaggerated proinflammatory cellular immune response [12], [32].

In the NMOSD group, no difference regarding gender was found between AQP4-Ab positive and negative patients (p = 0.23, Fisher’s exact test). 15 out 32 (47%) patients self-reported their ethic origin as afrodescendant in the NMOSD group, 9 out of 17 (53%) in the HAM/TSP group, and 7 out 15 (47%) in the HTLV-1+ group.

**Figure 2:**
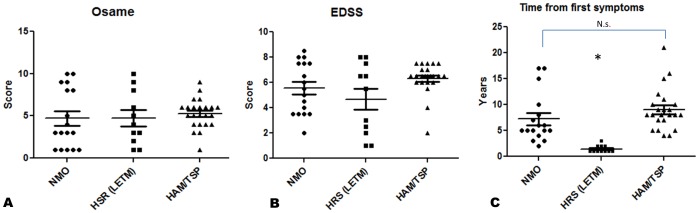
Disease duration and disability. (**A** and **B**) Disability scores as measured by the Osame scale (p = 0.52, Kruskal Wallis Test) and by the EDSS scores (p = 0.35, Kruskal Wallis Test) did not differ significantly between patients with established NMO, non-HTLV-1-associated LETM, and HAM/TSP. (**C**) The fact that the median Osame and EDSS scores did not differ between LETM and the other groups despite shorter disease duration in the LETM group reflects the rapid accumulation of disability in NMOSD as described before (p<0.0001, Kruskal Wallis Test. Dunn’s multiple comparison test did not demonstrate significant difference between NMO vs. HAM/TSP groups) [1], [2].

Regarding the median degree of disability, as measured by Osame scale (p = 0.47) and EDSS (p = 0.52), there were no statistically significant differences between the three groups (Figure 2). An Osame scale score > = 4 (unilateral walking aid needed) was found in 80% of the HAM/TSP patients, in 47% of the NMO patients, and in 54% of the LETM patients. An EDSS score ≥6.5 (constant bilateral assistance needed to walk at least 20 meters without resting) had 60% of the HAM/TSP patients, 41% of the NMO patients, and 45% of the LETM patients.

## Discussion

Our findings are relevant not only from a diagnostic but also from a pathophysiological point of view. HTLV-1 infection is highly prevalent among Brazilians, and patients with HAM/TSP can present with an acute/subacute form of longitudinally extensive myelitis, which can be confused with LETM lesions seen in NMOSD on MRI. Our results argue both against a role of antibodies to AQP4 in the pathogenesis of HAM/TSP and against an association between HTLV-1 infection and the development of AQP4-Ab. Moreover, the fact that antibodies to HTLV-1 were absent in all patients with NMOSD suggests that HTLV-1 is not a common trigger of acute attacks in patients with AQP4-Ab positive NMOSD, a disorder in which relapses are often preceded by infection, in populations with high HTLV-1 seroprevalence.

NMOSD and HAM/TSP are both highly prevalent in some global areas. In Japan and in Martinique, for example, NMOSD accounts for around 40% and 17.3% of cases of CNS demyelinating diseases, respectively, and these areas also feature a high prevalence of HTLV-1 infection [12], [18], [19]. In Brazil, around 30% of patients with CNS demyelinating diseases present with optic spinal symptoms and around 12% with strict NMO [20]. The seroprevalence of HTLV-1 has been reported to be 0.45% among volunteer blood-donors in Brazil in a nationwide survey [21]. Epidemiological studies indicate that NMOSD more commonly affects patients of non-Caucasian background [17], as do HTLV-1 infection. Similarly, a higher proportion of afrodescendants and Asians than Caucasians has been found among Brazilian HTLV-1 patients [22]; the latter fact has been discussed to reflect the African origin of HTLV-1, which is thought to be derived from primate T-lymphotropic virus (PTLV) and later spreading with the old population’s migratory pathway [12]. Accordingly, we found a high proportion of individuals with afrodescendant both in the NMOSD and in the HAM/TSP group in our study.

Regarding the patients’ epidemiological data, we found an older median age at onset in the HAM/TSP group compared to the NMOSD group in accordance with the literature, possibly reflecting the long latency of HTLV-1 infection [9], [29]. A female preponderance was found in all groups. While the higher proportion of females in the HAM/TSP group can be explained by the higher sexual transmission efficiency from men to women than from women to men [33], that observed in the NMOSD group is in line with the putative autoimmune aetiology of this disorder; female preponderance is a common characteristic of many autoimmune diseases [34].

The relative high EDSS and Osame scores in the NMOSD group as compared to the HAM/TSP group despite a shorter disease duration reflects the attack severity in NMOSD with poor recovery [1], [2], which is in contrast with the mostly progressive course of disease in HAM/TSP. The frequency and distribution of pain symptoms in the NMOSD group is in line with the literature [2], [35]. The relatively low frequency of CSF oligoclonal bands (29%) in our NMOSD patients is also in accordance with previous studies, which consistently demonstrated a lower frequency of CSF OCB when compared to multiple sclerosis patients [1], [36], [37].

In conclusion, our findings indicate that misdiagnosis of NMOSD as HAM/TSP is rare and support the view that LETM associated to either HAM/TSP or NMO are distinct disorders, which are likely to be pathogenetically unrelated.
